# Swimming pool attendance is related to asthma among atopic school children: a population-based study

**DOI:** 10.1186/s12940-015-0023-x

**Published:** 2015-04-15

**Authors:** Martin Andersson, Linnea Hedman, Gunnar Nordberg, Bertil Forsberg, Kåre Eriksson, Eva Rönmark

**Affiliations:** Department of Public Health and Clinical Medicine, Occupational and Environmental Medicine, Umeå University, S-90187 Umeå, Sweden; The OLIN Studies, Norrbotten County Council, Luleå, Sweden

**Keywords:** Asthma, Children, Swimming pools, Trichloramines, Sensitization

## Abstract

**Background:**

By-products of water disinfectants have been suggested to cause asthma, especially in atopic children. However, studies on indoor swimming pool attendance and asthma in children have presented conflicting results. The present study examined the relationship between indoor swimming pool attendance and asthma among sensitized and non-sensitized children aged 11-12 years.

**Methods:**

An extended ISAAC questionnaire was sent to the families of all children attending fifth or sixth grade, aged 11-12 years, in two municipalities in Northern Sweden in 2010. A total of 1866 participated (96% of those invited) in the questionnaire study and 1652 (89%) also participated in skin prick testing for 10 standard airborne allergens. Asthma was defined as physician-diagnosed asthma in combination with wheeze or use of asthma medication in the last 12 months. Current swimming pool attendance was reported as ≥1/week or <1/week. Logistic regression models were used for data analysis.

**Results:**

The prevalence of current asthma was 8.9% (10.0% of boys; 7.9% of girls) and 14% had attended indoor pools ≥1/week. Children currently attending swimming pools ≥1/week had an increased risk of current asthma. Stratified analyses for allergic sensitization adjusted for sex, parental smoking, parental asthma, and damp housing, showed a statistically significant association for current asthma only among sensitized subjects (OR 95% CI 1.90 1.09-3.32). No association was found between current pool attendance and wheeze, sensitization, rhinitis or eczema.

**Conclusions:**

The present study supports the proposed link between indoor swimming pool attendance and asthma in sensitized children.

## Background

Globally, asthma is the fourth most important cause of disability-adjusted life years (DALYs) in the 10–14 year age group [1]. Many environmental risk factors for childhood asthma have been identified, including traffic-related air pollution [2,3], early exposure to damp housing [4], and parental tobacco smoking [5-7]. Knowledge about environmental risk factors is important, as it may provide increased opportunities for prevention. Exposure to indoor swimming pools and water disinfectant by-products (DBPs), for example, trichloramines [8], has been suggested as a cause of asthma [9-11]. However, a review conducted in 2009 found that the evidence of an association was suggestive rather than conclusive [12]. In addition, recent studies show conflicting results [11,13-15].

Swimming has beneficial health effects, such as increased physical activity in children. However, if there is a causal relationship between exposure to DBPs and childhood asthma, failing to take precautionary steps may lead to new-onset asthma. This risk indicates that more research in this area is warranted [12]. In 2006, the second Obstructive Lung Disease in Northern Sweden (OLIN) pediatric cohort was established, and data regarding prevalence and risk factors for asthma, wheeze, and allergic sensitization have been reported [3,16-18]. In the 2010 follow-up of the cohort, a question on indoor swimming pool attendance was added to the questionnaire. The present study aimed to investigate the association between exposure to indoor swimming pool environments and asthma and allergy in a large and well-characterized cohort of 11-12 year old children.

## Methods

### The OLIN pediatric cohort II

The second OLIN pediatric cohort was recruited in 2006 and comprised 2585 children. The study area consisted of the municipalities of Luleå, Kiruna, and Piteå in northern Sweden. All children in the first and second grade of primary school, aged 7-8 years, were invited and their parents answered a questionnaire. The questionnaire [16] included the ISAAC (The International Study on Asthma and Allergy in Childhood) core questions on asthma symptoms and allergy [19]. Specific questions on environmental factors, heredity, physician-diagnoses, and medication for asthma and allergy were added [16].

The close collaboration between the OLIN study group and the schools in the study area contributed to a high participation rate, 96% [16,17]. The self-reported physician-diagnosis of asthma among 7-8 year old children were validated by an asthma questionnaire and pediatric assessment in the same study area, with a sensitivity of 70% and specificity of >99% [20].

### The study design

The cohort was re-surveyed in 2010 with all children, now in fifth and sixth grade, 11-12 years old, invited to participate. The children’s parents were asked to complete the same questionnaire as in 2006, and 95% participated (n = 2612). For the present study, only children in the municipalities of Luleå and Kiruna (n = 1866; 96% of invited) were included as skin prick tests (n = 1652; 89%) were only performed in these cities. In the 2010 questionnaire, a question on current indoor swimming pool attendance was added. This allowed a cross-sectional analysis to be performed on the association between attending indoor swimming pools and symptoms and diagnoses of asthma and allergy at age 11-12 years. The study was approved by the Regional Ethical Review Board at Umeå University, Sweden.

### Skin prick testing

Skin prick tests (SPT) following the European Academy of Allergy and Clinical Immunology (EAACI) method were performed in 2010 at the age of 11-12 years [21]. Ten standard airborne allergens were tested for (birch, timothy, mugwort, cat, dog, horse, two molds and two mites). A mean wheal diameter ≥3 mm for a specific allergen was regarded as a positive test. The SPT results has been validated against serum-IgE in this cohort [16].

### Definitions

*Current wheeze:* “Has your child had wheezing or whistling in the chest in the last 12 months?” [19]

*Physician-diagnosed asthma:* “Has your child been diagnosed by a physician as having asthma?” [16]

*Current medication*: Use of any kind of asthma medicine in the last 12 months [16].

*Current asthma: Physician-diagnosed asthma* and either *current wheeze* or *current medication* [16].

*Allergic sensitization:* Any positive SPT.

*Allergic rhinitis:* “Has the child during the last 12 months had sneezing, runny nose or nasal obstruction without having had a common cold?” [19]

*Parental smoking:* Father or mother smokes [16].

*Parental asthma:* Father or mother with asthma [16].

*Damp housing:* Previously or currently living in a damp building [16].

*Swimming pool attendance:* “How often do you go to an indoor swimming pool?”

The alternatives were: Never or a few times each year; some time every month; some time every week; and three times or more every week. The answers were dichotomized as ≥1/week and <1/week.

### Statistical analysis

Odds ratios (OR) with 95% confidence intervals (95% CI) were calculated in binary logistic regression analyses. These were performed for all children and separately for sensitized and non-sensitized children. Subsequent multivariate analyses were adjusted for sex, parental smoking, parental asthma, and damp housing. Comparisons of prevalence across groups were performed with Chi-square tests, or Mantel-Haenszel test for trend where appropriate. All analyses were executed in the SPSS software (v.22, Chicago, IL, USA).

## Results

The characteristics of the study population are presented in Table 1. The prevalence of allergic sensitization to at least one allergen was 39.9% and 10.9% had physician-diagnosed asthma, both significantly more common among boys. Current asthma was reported by 8.9%. The prevalence of currently attending indoor swimming pools were: never/some time every year 32.0%; some time every month 54.0%; some time every week 12.4%; three times a week or more 1.5%. Thus, 14.0% had attended an indoor swimming pool once a week or more.

**Table 1:** **Characteristics of the study population**

	**Sensitized children (n = 659) %**	**Non-sensitized children (n = 993) %**	**All children (n = 1866) %**	**Difference by sensitization status p-value**
Male sex	56.8	47.2	51.0	<0.001
Current wheeze	20.0	5.1	10.7	<0.001
Physician-diagnosed asthma	19.7	5.9	10.9	<0.001
Current asthma	17.1	4.1	8.9	<0.001
Current asthma medication	25.0	7.8	13.8	<0.001
Allergic sensitization	-	-	39.9	-
Current rhinitis	46.1	10.4	23.3	<0.001
Physician-diagnosed rhinitis	25.5	2.3	11.2	<0.001
Current eczema	30.8	16.8	21.2	<0.001
Physician-diagnosed eczema	24.7	10.2	15.3	<0.001
Parental asthma	31.0	20.7	24.0	<0.001
Parental smoking	20.5	21.7	21.1	0.545
Damp housing	11.9	9.6	10.8	0.141
Swimming pool attendance ≥1/week	15.1	13.7	14.0	0.436

Prevalence (%) of conditions, risk factors and environmental factors in the OLIN pediatric cohort 2010. Data are presented separately for all children in the study population and the children in the study population by sensitization status. P value calculated by Chi-square test.

In the bivariate analyses, indoor swimming pool attendance was significantly associated with both physician-diagnosed and current asthma (OR 1.49, 95% CI 1.02–2.18 and OR 1.54, 95% CI 1.02–2.31, respectively), but not with current wheeze (OR 1.02, 95% CI 0.67–1.56). There was no association between indoor swimming and allergic sensitization (OR 1.12 95% CI 0.84-1.48). When stratified according to sensitization status, both physician-diagnosed asthma and current asthma among sensitized children were significantly associated with indoor swimming (OR 2.01, 95% CI 1.24–3.25 and OR 2.08, 95% CI 1.26–3.43, respectively), while among non-sensitized children no significant associations were found.

In order to assess a possible-dose response trend, the exposure variable (currently attending indoor swimming pools) was classified into three groups: never/some time every year; some time every month and some time every week or more. A significant association between current asthma and the frequency of attending indoor swimming pools was found (Table 2).

**Table 2:** **The risk of asthma and current wheeze in relation to frequency of indoor swimming pool attendance**

	**Currently attending indoor swimming pools**			
	**Never or sometime/year (n = 883)**	**Some time/month (n = 1387)**	**Every week or more (n = 323)**	P*
Physician-diagnosed asthma	9.3%	11.0%	14.7%	0.027
Current asthma	7.9%	8.7%	12.4%	0.065
Current wheeze	9.3%	11.4%	10.8%	0.332

*Test for trend conducted by Mantel-Haenszel test.

Results presented as the prevalence of asthma and wheeze, respectively, by frequency of attending indoor swimming pools.

### Adjusted analyses

**Figure 1:**
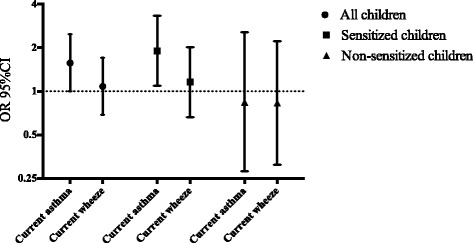
Attending indoor swimming pools ≥1/week in relation to current asthma, and current wheeze, respectively. Risk expressed as adjusted odds ratios (OR 95% CI), calculated by multiple logistic regression and adjusted for sex, parental asthma, parental smoking and damp housing. The results are presented for all children in the study population and for sensitized and non-sensitized children separately.

The analyses of indoor swimming pool attendance in relation to asthma, rhinitis and eczema were adjusted for sex, parental smoking, parental asthma, and damp housing. The results did not change markedly from those of the bivariate analyses and none of the covariables adjusted for were associated with swimming pool attendance. These analyses were also conducted separately for sensitized and non-sensitized children (Figure 1 and Table 3). Testing for statistical interaction between sensitization and swimming pool attendance was performed and showed borderline statistical significance (p 0.056).

**Table 3:** **The risk of asthma and other conditions in relation to indoor swimming pool attendance**

	**Sensitized children (n = 659)**	**Non-sensitized children (n = 993)**	**All children (n = 1866)**
Physician-diagnosed asthma	1.93 1.13-3.31	1.04 0.44-2.45	1.55 1.02-2.36
Current asthma	1.90 1.09-3.32	0.84 0.28-2.57	1.57 1.00-2.48
Current wheeze	1.16 0.66-2.02	0.83 0.31-2.22	1.08 0.69-1.70
Physician-diagnosed rhinitis	1.21 0.73-2.00	0.32 0.04-2.39	1.05 0.68-1.61
Current rhinitis	1.21 0.76-1.91	1.62 0.93-2.82	1.29 0.94-1.77
Physician-diagnosed eczema	1.19 0.72-1.99	0.74 0.37-1.47	1.01 0.69-1.48
Current eczema	0.68 0.40-1.15	0.99 0.60-1.64	0.88 0.62-1.24

The analyses have been adjusted for sex, parental asthma, parental smoking and damp housing. The exposure variable was currently attending indoor swimming pools ≥1/week.

Risk estimates express as Odds Ratios (OR) with 95% confidence intervals (CI). Data are presented separately for all children in the study population and the children in the study population by sensitization status.

In sensitized children, indoor swimming pool attendance was significantly associated with physician-diagnosed and current asthma (OR 1.93, 95% CI 1.13–3.31 and OR 1.90, 95% CI 1.09–3.32, respectively). Current wheeze was not significantly associated with attending swimming pools more than once per week (OR 1.16, 95% CI 0.66–2.02).

Among non-sensitized children there were no significant associations between pool attendance and physician-diagnosed asthma (OR 1.04, 95% CI 0.44–2.45), current asthma (OR 0.84, 95% CI 0.28–2.57), or current wheeze (OR 0.83, 95% CI 0.31–2.22) (Table 3).

Indoor swimming pool attendance were not associated with current rhinitis (OR 1.29, 95% CI 0.94–1.77), or physician-diagnosed rhinitis (OR 1.05, 95% CI 0.68–1.61). In addition, there was no association between swimming pool attendance and current eczema (OR 0.88, 95% CI 0.62–1.24), or physician-diagnosed eczema (OR 1.01, 95% CI 0.69–1.48). There were no significant correlations between swimming pool attendance and neither rhinitis nor eczema when stratified according to sensitization status.

## Discussion

The present study found an increased risk of asthma in sensitized children currently attending indoor swimming pools once a week or more. The increased risk was also present after adjusting for sex, parental smoking, parental asthma, and damp housing. If this finding represents a true causality, consideration of changes in indoor swimming pool environments are an important area for public health interventions. Preventing asthma among sensitized children is warranted, as sensitization is a strong risk factor for persistence of asthma in adolescence [22,23].

The chlorinated pool hypothesis is controversial, and the results of previous studies are considered inconclusive [12]. There are few large population-based studies on the risk of childhood asthma in relation to indoor swimming pool attendance [12-14]. The present study is one of the first studies to investigate this topic in the Nordic countries [24]. An increased risk of asthma has been found in a smaller study where the exposure to trichloramine was estimated to 0.3–0.5 mg/m^3^ [10]. Conversely, large population-based studies have not observed any respiratory health effects to date, and these studies have neither found any interaction between swimming pool attendance and sensitization on asthma [13-15]. In one of these studies [13] trichloramine was measured, but there were lower trichloramine levels than what has previously been measured in northern Sweden [25].

The chlorinated pool hypothesis suggests that by-products of water disinfectants, especially trichloramine, may cause asthma by damaging the respiratory epithelium. This epithelial damage could be facilitated by an existent sensitization to aeroallergens or increase the risk of sensitization in non-atopic children by reducing the protective properties of the pulmonary epithelium [9,10,26-28]. No change in biomarkers of epithelial integrity was found among non-allergic subjects exposed to pool environments [25], indicating that an interaction may be required in order to cause an effect.

In the present study, the association between asthma and indoor swimming pool attendance was restricted to sensitized children. This finding was interesting, as atopic children attending or not attending pool environments have a strong and consistent risk factor for asthma in epidemiological studies; namely, allergic sensitization. However, a Belgian study reported an increased risk of childhood asthma only in atopic children with a total IgE of >100 kIU/L [10]. A previous study from Sweden found no effect on biomarkers of epithelial integrity among 37 non-allergic volunteers with low IgE values after exposure to pool environments [25]. These observations align with the present results. In a study by Kohlhammer et al. there was an increased risk of hay fever in an adult population from school age exposure [29]. In addition, eczema has been linked to swimming pool attendance [13,30]. While these findings support an interaction between pool exposure and sensitization, caution is warranted when interpreting the results, as the testing for statistical interaction was only borderline significant and as there are inconsistent results on lung epithelium biomarkers and asthma after swimming, also among atopic children [13-15,31].

In the present study, rhinitis and eczema were not significantly associated with swimming pool attendance. A possible explanation is that rhinitis and eczema are more probable outcomes in other age groups; rhinitis in adulthood [29], and eczema in early childhood [30]. The negative association between eczema in atopic children and swimming pool attendance could also indicate that these children avoid indoor swimming pools because of the well-known negative impact of water on eczema. However, Font-Ribera et al. found an increased risk of eczema in a similar age group as the present study [13]. In our study, although the risk was slightly elevated, current wheeze was not significantly associated with indoor swimming pool attendance. Current wheeze is often covariate to physician-diagnosed asthma in epidemiological studies in childhood, but it is less specific than a physician-diagnosis of asthma and may also represent other and temporary conditions, for example, respiratory infections. The lack of association between current wheeze and swimming pool attendance in the present study could be the result of such a dilution effect. Another possible explanation for these findings is that if swimming pool attendance causes asthma and wheezing at an early age [24], children at 12 years of age may have remitted from wheezing while retaining the asthma diagnosis into school age. However, this is less likely given the present study found an equally strong association between pool attendance and current asthma (i.e., asthma with current wheeze or current medication) as between pool attendance and physician-diagnosed asthma.

The exposure assessment in our study was based on parental reports from the questionnaire. To allow statistical tests to be performed, the reported exposure to indoor swimming pools was dichotomized with attendance of ≥ 1 time/week as the exposed group. This aimed to designate the exposed group in such a way that it included about one tenth of the population. Swimming once a week or more has also been used as an exposure variable in other population-based studies [14], although it should be remembered that in our study, swimming was specified as in indoor pools. While no measurements were done in the present study, a mean level of 0.21 mg/m^3^ of trichloramine (ranging from 0.09–0.32) has been reported from other indoor pools in Northern Sweden [25]. A strong association between swimming pool attendance and asthma among sensitized children was found, despite the lack of a detailed exposure assessment. However, our analyses regarding frequency of swimming pool attendance results indicate a dose-response effect. This means that the risk may be underestimated. In future studies, better exposure assessments are needed; for example objective measurements of trichloramines and other chemicals as well as obtaining detailed exposure histories.

The present results are strengthened by the population-based study design and the large number of participating children. Further, the participation rate of 96% is high and ensures that the study sample was representative. The self-reported physician-diagnose of asthma was validated [20] and the questionnaire was based on the well-established and valid ISAAC questions and methodology [19]. The children are well characterized as to possible confounding factors and co-morbidities [16]. However, the information on socioeconomic status, e.g. family income, was limited because such questions was considered to risk a lower participation rate by being perceived as intrusive. Instead, we choose to use damp housing as a proxy variable for socioeconomic status. Further, the socioeconomic differences in northern Sweden are relatively small. Also other unmeasured confounding, e.g. vehicle traffic exposure or physical activity, cannot be excluded. However, such possible factors still seem to mainly affect atopic children according to our results. For example, there could be an association between living in a city with much vehicle traffic and having a swimming pool establishment within close distance. In this study, we do not have the data to adjust for this possible cofounding factor. A previous study in the same area however found no association between vehicle traffic and asthma among atopic children [3].

There are two main limitations in the present study. First, data collection relied on self-reported exposure assessments of indoor pool attendance and second, the study design was cross-sectional. Therefore, completely ruling out reverse causation or recall bias in our results are not possible, especially so as there was no information on historical exposure to swimming pools, e.g. infant swimming. However, the association between asthma and swimming pool attendance was only seen among sensitized children, so is hard to dismiss as being caused by reverse causation or recall bias. If physicians recommend their patients with asthma to attend swimming pools as a way of exercising, it is unlikely that they would consistently only make this recommendation to atopic children. It is also unlikely that only atopic children with asthma would over-report their swimming pool attendance. While public awareness on asthma in relation to swimming pools cannot be excluded, recommendations on swimming pool exercise to asthmatic children is, in our experience, not common in Sweden.

## Conclusions

Our study supports the proposed link between indoor swimming pool attendance and asthma in sensitized children. Future studies should explore the risk for atopic and non-atopic children separately.

## Abbreviations

DALYS: Disability adjusted life years
DBP: Disinfection by-products
EAACI: European Academy of Allergology and Clinical Immunology
ISAAC: The International Study on Asthma and Allergies in Childhood
OLIN: The obstructive lung disease in Northern Sweden studies
OR: Odds ratio
SPT: Skin prick test

## References

[1] Gore FM, Bloem PJ, Patton GC, Ferguson J, Joseph V, Coffey C (2011). Global burden of disease in young people aged 10-24 years: a systematic analysis. Lancet.

[2] McConnell R, Islam T, Shankardass K, Jerrett M, Lurmann F, Gilliland F (2010). Childhood incident asthma and traffic-related air pollution at home and school. Environ Health Perspect.

[3] Andersson M, Modig L, Hedman L, Forsberg B, Ronmark E (2011). Heavy vehicle traffic is related to wheeze among schoolchildren: a population-based study in an area with low traffic flows. Environ Health.

[4] Tischer CG, Hohmann C, Thiering E, Herbarth O, Muller A, Henderson J (2011). Meta-analysis of mould and dampness exposure on asthma and allergy in eight European birth cohorts: an ENRIECO initiative. Allergy.

[5] Genuneit J, Weinmayr G, Radon K, Dressel H, Windstetter D, Rzehak P (2006). Smoking and the incidence of asthma during adolescence: results of a large cohort study in Germany. Thorax.

[6] Bjerg A, Hedman L, Perzanowski M, Lundback B, Ronmark E (2011). A strong synergism of low birth weight and prenatal smoking on asthma in schoolchildren. Pediatrics.

[7] Hedman L, Bjerg A, Sundberg S, Forsberg B, Ronmark E (2011). Both environmental tobacco smoke and personal smoking is related to asthma and wheeze in teenagers. Thorax.

[8] Zwiener C, Richardson SD, DeMarini DM, Grummt T, Glauner T, Frimmel FH (2007). Drowning in disinfection byproducts? Assessing swimming pool water. Environ Sci Tech.

[9] Bernard A, Nickmilder M, Voisin C, Sardella A (2009). Impact of chlorinated swimming pool attendance on the respiratory health of adolescents. Pediatrics.

[10] Bernard A, Carbonnelle S, de Burbure C, Michel O, Nickmilder M (2006). Chlorinated pool attendance, atopy, and the risk of asthma during childhood. Environ Health Perspect.

[11] Voisin C, Sardella A, Marcucci F, Bernard A (2010). Infant swimming in chlorinated pools and the risks of bronchiolitis, asthma and allergy. Eur Respir J.

[12] Weisel CP, Richardson SD, Nemery B, Aggazzotti G, Baraldi E, Blatchley ER (2009). Childhood asthma and environmental exposures at swimming pools: state of the science and research recommendations. Environ Health Perspect.

[13] Font-Ribera L, Kogevinas M, Zock JP, Nieuwenhuijsen MJ, Heederik D, Villanueva CM (2009). Swimming pool attendance and risk of asthma and allergic symptoms in children. Eur Respir J.

[14] Font-Ribera L, Villanueva CM, Nieuwenhuijsen MJ, Zock JP, Kogevinas M, Henderson J (2011). Swimming pool attendance, asthma, allergies, and lung function in the Avon longitudinal study of parents and children cohort. Am J Respir Crit Care Med.

[15] Jacobs JH, Fuertes E, Krop EJ, Spithoven J, Tromp P, Heederik DJ (2012). Swimming pool attendance and respiratory symptoms and allergies among Dutch children. Occup Environ Med.

[16] Ronmark E, Bjerg A, Perzanowski M, Platts-Mills T, Lundback B (2009). Major increase in allergic sensitization in schoolchildren from 1996 to 2006 in northern Sweden. J Allergy Clin Immun.

[17] Bjerg A, Sandstrom T, Lundback B, Ronmark E (2010). Time trends in asthma and wheeze in Swedish children 1996-2006: prevalence and risk factors by sex. Allergy.

[18] Andersson M, Bjerg A, Forsberg B, Lundback B, Ronmark E (2010). The clinical expression of asthma in schoolchildren has changed between 1996 and 2006. Pediatr Allergy Immunol.

[19] Asher MI, Keil U, Anderson HR, Beasley R, Crane J, Martinez F (1995). International Study of Asthma and Allergies in Childhood (ISAAC): rationale and methods. Eur Respir J.

[20] Ronmark E, Jonsson E, Platts-Mills T, Lundback B (1999). Different pattern of risk factors for atopic and nonatopic asthma among children–report from the Obstructive Lung Disease in Northern Sweden Study. Allergy.

[21] Dreborg S (1989). Skin-tests used in Type-I allergy testing position paper. Allergy.

[22] Sears MR, Greene JM, Willan AR, Wiecek EM, Taylor DR, Flannery EM (2003). A longitudinal, population-based, cohort study of childhood asthma followed to adulthood. N Engl J Med.

[23] Andersson M, Hedman L, Bjerg A, Forsberg B, Lundback B, Ronmark E (2013). Remission and persistence of asthma followed from 7 to 19 years of age. Pediatrics.

[24] Nystad W, Haberg SE, London SJ, Nafstad P, Magnus P (2008). Baby swimming and respiratory health. Acta Paediatr.

[25] Nordberg GF, Lundstrom NG, Forsberg B, Hagenbjork-Gustafsson A, Lagerkvist BJ, Nilsson J (2012). Lung function in volunteers before and after exposure to trichloramine in indoor pool environments and asthma in a cohort of pool workers. BMJ Open.

[26] Lagerkvist BJ, Bernard A, Blomberg A, Bergstrom E, Forsberg B, Holmstrom K (2004). Pulmonary epithelial integrity in children: relationship to ambient ozone exposure and swimming pool attendance. Environ Health Perspect.

[27] Bernard A, Carbonnelle S, Michel O, Higuet S, De Burbure C, Buchet JP (2003). Lung hyperpermeability and asthma prevalence in schoolchildren: unexpected associations with the attendance at indoor chlorinated swimming pools. Occup Environ Med.

[28] Bernard A, Voisin C, Sardella A (2011). Con: respiratory risks associated with chlorinated swimming pools: a complex pattern of exposure and effects. Am J Respir Crit Care Med.

[29] Kohlhammer Y, Doring A, Schafer T, Wichmann HE, Heinrich J (2006). Swimming pool attendance and hay fever rates later in life. Allergy.

[30] Chaumont A, Voisin C, Sardella A, Bernard A (2012). Interactions between domestic water hardness, infant swimming and atopy in the development of childhood eczema. Environ Res.

[31] Font-Ribera L, Kogevinas M, Zock JP, Gomez FP, Barreiro E, Nieuwenhuijsen MJ (2010). Short-term changes in respiratory biomarkers after swimming in a chlorinated pool. Environ Health Perspect.

